# Effects of photobiomodulation on re-epithelialization of burn wound: protocol for a randomized controlled trial

**DOI:** 10.1186/s13063-022-06505-4

**Published:** 2022-07-19

**Authors:** Silvana Cristina de Araújo Pereira Venceslau, Júlia Lacet Silva Ferreira, Renata Maria Freire Barros, Ronny Marcos de Morais, Heleodório Honorato dos Santos, Valéria Mayaly Alves de Oliveira, Palloma Rodrigues de Andrade

**Affiliations:** 1 Postgraduate Program in Physical Therapy/Health Sciences Center/Federal University of Paraíba, Campus I - Cidade Universitária, João Pessoa, PB 58059-900 Brazil; 2 Healthy Sciences Centre/Fereal University of Paraíba, João Pessoa, Brazil

**Keywords:** Photobiomodulation, Healing, Burns, Randomized controlled trial

## Abstract

**Background:**

Burns are a global public health problem and cause approximately 180,000 deaths annually, mainly in low- and middle-income countries. Topical antibiotics and occlusive dressing are standard treatments for burns not requiring a skin graft. However, physiotherapy has low-cost phototherapeutic and electrophysical resources (e.g., light-emitting diode [LED]) that may accelerate burn healing. This study aims to compare the re-epithelialization rate of second-degree burns, pain, pruritus, skin temperature, quality of wound healing, and scar quality and mobility among individuals undergoing treatment with red LED, infrared LED, and simulated photobiomodulation.

**Methods:**

This is a double-blinded, three-arm parallel-group, randomized controlled superiority trial. Individuals of both sexes, aged over 18 years, and with second-degree burns will be included. The sample will be divided into three groups of 13 individuals: two will receive LED therapy (red or infrared) and one placebo. Pain, pruritus, skin temperature, and wound size will be assessed daily. Interventions will take place until complete healing, when scar mobility and quality will be evaluated. Data will be presented as mean and 95% confidence interval and analyzed using mixed linear models.

**Discussion:**

This randomized controlled trial has minimal risk of bias and intends to identify the ideal type, procedures, and doses of photobiomodulation to heal burns, which are not standardized in clinical practice. Positive results will allow the implementation of the technique in burn and wound guidelines.

**Trial registration:**

Brazilian Clinical Trials Registry (ReBEC) RBR-8bfznx6. Registered on October 13, 2021

## Administrative information

Note: The numbers in curly brackets in this protocol refer to SPIRIT checklist item numbers. The order of the items has been modified to group similar items (see http://www.equator-network.org/reporting-guidelines/spirit-2013-statement-defining-standard-protocol-items-for-clinical-trials/).

**Table:** 

Title {1}	Effects of photobiomodulation on re-epithelialization of burn wound: protocol for a randomized controlled trial
Trial registration {2a and 2b}	Brazilian Clinical Trials Registry – ReBEC; Registration number - RBR-8bfznx6; [registered on 10-13-2021]
Protocol version {3}	Version 1 of 10-13-2021
Funding {4}	This research is funded by the Federal University of Paraiba (public notice n.03/2021) and by the Research Support Foundation of the State of Paraiba (universal public notice).
Author details {5a}	S.C.A.P.V. [Federal University of Paraiba, Brazil], J.L.S.F. [Federal University of Paraiba, Brazil], R.M.F.B. [Federal University of Paraiba, Brazil], R.M.M. [Federal University of Paraiba, Brazil], H.H.S. [Federal University of Paraiba, Brazil], V.M.A.O. [Federal University of Paraiba, Brazil], P.R.A. [Federal University of Paraiba, Brazil] S.C.A.P.V and P.R.A conceived of the study. J.L.S.F., R.M.F.B., R.M.M initiated study design and H.H.S. and V.M.A.O helped with implementation. V.M.A.O and P.R.A provide statistical expertise in clinical trial design. All authors contributed to refinement of the study protocol and approved the final manuscript.
Name and contact information of the trial sponsor {5b}	Investigator-initiated clinical trial; P.R. Andrade (Principal Investigator) Campus I - Cidade Universitária - João Pessoa - Paraíba - Brazil – Postal Code: 58059-900 Fone: +558332167183. palloma@ccs.ufpb.br
Role of sponsor {5c}	This is an investigator-initiated clinical trial. This funding source had no role in the study design and will not have any role during its execution, analyses, data interpretation, or decision to submit the results.

## Introduction

### Background and rationale {6a}

Burns are a global public health problem that causes approximately 180,000 deaths annually, mainly in low- and middle-income countries [1]. The Brazilian government estimates about one million burning accidents per year, of which 2500 evolve to death [2]. Most burned individuals are of working age and generate high financial costs with physical and psychological treatments [3]. Burns heal within 7 to 21 days, depending on depth, patient age, associated diseases, medications, and lifestyle. Open wounds increase the risk of infection and absence from work [4–7].

Improvements in the treatment of severe burns decreased mortality and increased the need for rehabilitation [3, 8, 9]. Although 97% of patients admitted to burn centers survive, non-fatal burns lead to morbidity, psychosocial damage, scar contractures, pruritus, and deformities [10]. In this context, electrophysical resources, such as photobiomodulation, may improve symptoms and functionality during burn healing [11, 12].

Photobiomodulation has been used in animal experiments with promising results. Ideal wavelengths for burn healing are in red and infrared spectra; the former presents better results in cutaneous tissue and the latter in deeper tissues. Energy density ranging from 3 to 6 J/cm^2^ may also be effective for integument healing [13, 14]. Despite this, few clinical trials were published using low-level laser therapy in humans [15–17]. Moreover, only one study analyzed photobiomodulation with a light-emitting diode (LED) of 658 nm for burn healing. The authors included five individuals with bilateral burns (contralateral limb served as a control) and concluded that the limbs on the irradiated side healed faster than the non-irradiated side [14].

Photobiomodulation with LED is a non-invasive, low-cost, and highly viable treatment. Considering the scarcity of studies, we aimed to compare the re-epithelialization of second-degree burns using LED photobiomodulation treatment. We will also compare pain, pruritus, skin temperature, quality of wound healing, and scar quality and mobility among patients receiving treatment with red LED, infrared LED, and simulated photobiomodulation.

### Objectives {7}

#### Research hypothesis

LED will increase the re-epithelialization rate, decrease healing time and pain, and change the local temperature compared with simulated photobiomodulation.

### Study objectives

#### Primary objective

The primary objective is to determine whether LED photobiomodulation is superior to simulation to increase the re-epithelialization rate and decrease the healing time of burn wounds.

#### Secondary objective

The secondary objective is to determine whether LED photobiomodulation is superior to simulation to decrease pain and change the temperature of burn wounds.

### Trial design {8}

**Fig. 1:**
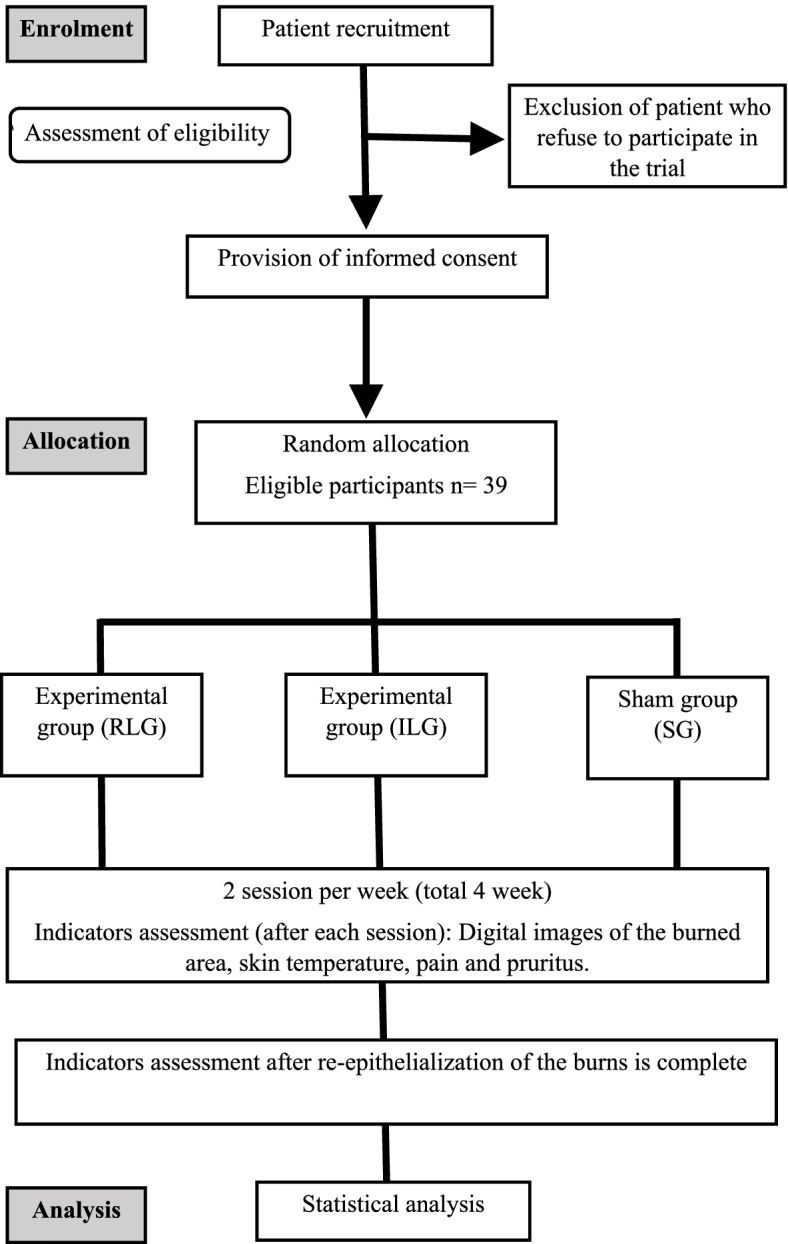
Study flowchart

This double-blinded, three-arm parallel-group, randomized, controlled, superiority trial (1:1:1) will be performed according to the Standard Protocol Items: Recommendations for Interventional Trials (SPIRIT) (Fig. 1).

## Methods: participants, interventions, and outcomes

### Study setting {9}

The study will be conducted from February 2022 to December 2023. Participants will be referred to the laboratory of the League of Studies in Dermatofunctional Physiotherapy (Department of Physiotherapy, Federal University of Paraíba, Brazil) from urgency and emergency services, basic health units of the city of João Pessoa, and the Hospital de Trauma Senador Humberto Lucena. Evaluations, interview for eligibility, data collection, and application of treatment protocols will also be conducted in the laboratory.

### Eligibility criteria {10}

We will include participants of both sexes who suffered second-degree burns, aged over 18 years, with good mental health (assessed using the Mini-Mental Status Exam) [18], without pre-existing metabolic diseases or infection in injured tissues, not under pharmacological treatment that alters the healing process (e.g., corticosteroids, chemotherapy, or radiotherapy), and who agree to participate in the research by signing the informed consent form.

Participants will be excluded if they develop dermatological diseases or infectious processes or wish to withdraw from the study for bioethical reasons.

### Who will take informed consent? {26a}

All participants will be informed about the evaluation and treatment procedures during the first interview, which will occur after referral to the laboratory and before randomization. They must sign two versions of the informed consent form to participate (one copy will remain with the participant), following Resolution 466/12 of the National Health Council and the Declaration of Helsinki.

### Additional consent provisions for collection and use of participant data and biological specimens {26b}

By signing the informed consent form, participants will allow us to contact their health professionals to inform them about participation and unexpected findings important to the health of the participant. They will also allow the research team to record one consultation with the health professional, request medical information from their files, and share data with competent authorities if needed. The informed consent form will have an item about the agreement to store and use the personal information of participants for future research. Biological specimens will not be collected for this trial.

### Interventions

#### Explanation for the choice of comparators {6b}

Although LED-sham generates similar sensations to active LED, its different effects will allow verifying whether LED (infrared or red) will affect the re-epithelialization rate. This similarity also ensures the blinding of participants regarding allocation and procedures.

#### Intervention description {11a}

All protocols will be performed using the Endophoton Esthetic Multi equipment (KLD - Brazil), calibrated according to the manual of the manufacturer (Amparo, São Paulo, Brazil). During application in all groups, the researcher and individuals will wear glasses with an eye protection filter for light radiation (suitable for applicators with wavelength between 415 and 904 nm) with minimum attenuation of 95%. The treatment protocol will be performed with individuals in dorsal decubitus and exposing the area to be treated. Photobiomodulation will last until complete re-epithelialization without a predetermined number of sessions, and duration of each session will occur according to wound size.

##### Red light experimental group (RLG)

Red photobiomodulation will be performed using a red LED pen with a wavelength of 658 nm and power of 12 × 40 mW (12 LEDs, each with an energy density of 3 J per point). The pen will be positioned perpendicular to the area to be treated and at the shortest distance without contacting the area. Points receiving therapy will be interspaced by 2 cm.

##### Infrared light experimental group (ILG)

Infrared photobiomodulation will be performed using the LED pen with a wavelength of 858 nm and power of 12 × 50 mW.

##### Sham group (SG)

The equipment will be programmed to emit as little energy as possible (i.e., no therapeutic results).

#### Criteria for discontinuing or modifying allocated interventions {11b}

Participants who present adverse effects due to the intervention protocol, complain of aggravation of the burn wound, or request alteration or withdrawal of the intervention will be discontinued or have their protocols modified.

#### Strategies to improve adherence to interventions {11c}

Attrition will be considered in case of missing two consecutive or three alternating sessions, inability to complete assessments after treatment and follow-up, and developing any disabling condition preventing the individual from participating in the study. Regarding adherence strategies, flexible schedules will be offered, and participants will be contacted by telephone to confirm evaluation dates. Additional measures to prevent dropouts will also be applied, including periodic assessments of satisfaction with treatment, discussion of difficulties in continuing treatment (e.g., logistics of visits to the laboratory or home care), and attempts to resolve and avoid potential problems affecting adherence. Furthermore, guidance regarding wound care and treatment will also be offered to increase adherence.

#### Relevant concomitant care permitted or prohibited during the trial {11d}

Treatments concomitant to the study will be allowed if not related to changes in research outcomes (e.g., simple dressing or hydration of the intact skin near the edge of the wound).

#### Provisions for post-trial care {30}

Provisions for post-trial care are not applicable in this study due to the nature of the intervention. All harms and adverse events will be properly managed. Participants will be referred to the respective referral services in case of health problems due to study procedures, and specialized medical assistance will be offered at no cost.

### Outcomes {12}

Participants will initially undergo an anamnesis to identify the presence of diabetes or other diseases interfering with biophysical aspects of the skin or characteristics of the burn. Evaluations will be conducted by a trained external evaluator blinded to allocation.

#### Primary outcome measures

##### Re-epithelialization rate

The percentage of healed skin tissue (re-epithelialization rate) and the number of days between the start of treatment and total healing process will be assessed using digital images of the burned region. These images will be captured daily from before treatment to follow-up of the re-epithelialization process. A digital camera (Sony Cyber-Shot Dsc-W800, 20.1 megapixels, Brazil) will be positioned on a tripod, parallel to the ground and perpendicularly fixed at a distance of 30 cm from the area to be treated. If the participant cannot stand, images will be captured on a stretcher assuring the same distance and a 90° angle between the area and the camera. The photograph will be captured on a white background with indirect artificial lighting.

Re-epithelialization rate will be assessed by counting the days until complete healing and monitoring the wound area during protocols (ImageJ® software, version 1.36 b). The crust area on the wound edge will be measured using digital image analysis, according to Gabison et al. [19]. The software converts the selected area to cm^2^ by associating the known dimension of the photographed object, area of interest, and the respective number of pixels. Healing will be assessed using a glossary of healing evolution based on the pressure ulcer assessment scale proposed by Bates-Jensen, Vredevoe, and Brecht [20].

#### Secondary outcome measures

##### Skin temperature

Thermographic evaluation will be conducted at the League of Studies in Dermatofunctional Physiotherapy of the Federal University of Paraíba, with room temperature between 22 and 24 °C and relative humidity of 43.0 ± 0.1% (monitored by thermohydrometer).

Participants will be previously instructed to avoid eating foods that alter thermogenesis, using body creams or oils, and wearing clothes that expose the region to be evaluated. Participants will also undergo a 15-min acclimatization to adapt to room temperature. Images will be taken with participants in orthostatic position. Thermograms will be obtained using a FlirOne camera (FLIR Systems, Inc., Wilsonville, OR) with an IR resolution of 120 × 160 pixels, temperature range from − 20 to 120 °C, and thermal sensitivity of 0.1°C. The camera will be fixed on a tripod parallel to the ground, with height adjusted to create a 90° angle with the region of interest, and at a distance of 30 cm. The distance from the camera to the area will be measured using a ruler fixed by the metallic support. If the volunteer cannot stand, images will be captured on a stretcher using the same metallic support to assure distance and angle between the area and the camera. FLIR tools software will process and analyze thermographic images.

##### Pain and local pruritus 

Pain intensity in the treated area will be assessed daily, before therapy, and until the end of treatment using the visual analog scale (VAS). This scale consists of a graduated line with scores ranging from 0 to 10 (0 represents “no pain” and 10 “worst pain possible”). Pain using VAS can be classified as mild (up to 3 points), moderate (from 4 to 7 points), or severe (> 7 points). Individuals will be asked to indicate the number on the scale that represents pain intensity.

The intensity of pruritus will also be assessed daily, before therapy, and until the end of treatment using the Itch Severity Scale, translated and validated to Brazilian Portuguese [21] and adapted for individuals with burn wounds. The scale consists of seven questions (description, frequency, intensity, extension of pruritus, and how pruritus affects sleep, mood, and sexual activity or desire), ranging from zero to one. The total score is obtained by the sum of all questions multiplied by a correction factor (×3) and ranges from 0 (no pruritus) to 21 points (most severe pruritus); minimal important difference is 2 points.

##### Scar mobility in the burned area 

After complete healing, an adheremeter will be positioned (without direct contact) in the central point of the scar most adhered to the skin. The skin will be stretched by centrifugation in four orthogonal directions (caudal, rostral, right, and left sides). Maximum distension will be read on the instrument after each traction. After releasing the tension, the researcher will observe if the landmark will return to the starting point; if not, the measurement will be repeated. This procedure will also be performed at the same location on the contralateral limb [22].

##### Quality of wound healing

The Vancouver scale will assess the quality of wound healing. It analyzes aspects of healing (e.g., vascularization, pigmentation, flexibility, and scar height) and presents a total score ranging from 0 (normal) to 13 (poor).

#### Participant timeline {13}

**Fig. 2:**
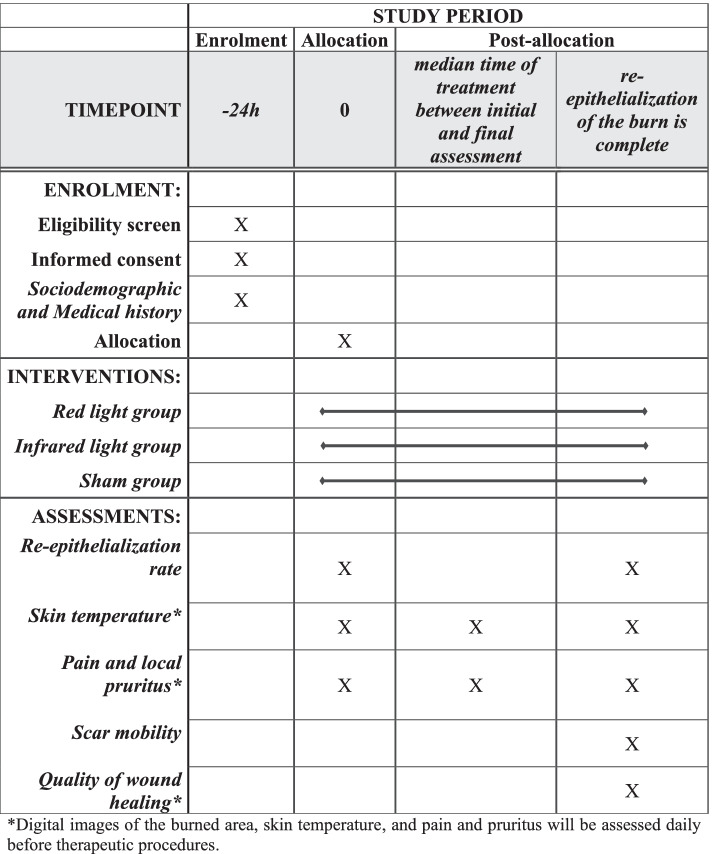
Participant timeline

Figure 2 represents an overview of the study design and main procedures.

Sociodemographic data, characteristics of the burn (i.e., type of accident, causal agent, and percentage of body surface affected), when the burn happened, and pharmacological treatment received will be registered on an evaluation form. Participants will be instructed to maintain standard care. A trained researcher will assess and perform therapeutic procedures five times a week, once daily. Wound healing rate, pain, pruritus, skin temperature, mobility restriction, and scar quality will be analyzed.

Digital images of the burned area, skin temperature (infrared image), and pain and pruritus will be assessed daily before therapeutic procedures. Only three assessments will be analyzed: initial (before treatment), final (when re-epithelialization of the burn is complete), and one assessment between initial and final assessments (median time of treatment between initial and final assessment). After treatment and total wound closure, mobility and scar quality will be assessed using the adheremeter and Vancouver Scale, respectively.

#### Sample size {14}

Randomized controlled trials conducted with participants with burn wounds are scarce, hindering the calculation of the effect size. Thus, a conservative effect size (0.5), 80% power, and 95% probability (*α* = 0.05) were used for the primary outcome (re-epithelialization rate), generating a sample size of 30 participants (three groups of ten participants). The sample size for repeated measures ANOVA was calculated using the G* Power 3.1.0 software (Christian-Albrechts-Universitat, Kiel, Germany). Considering a possible sample loss of 30%, we intend to recruit 39 participants.

#### Recruitment {15}

The research will be widely disclosed using social media, local TV, radio stations, and announcements in health units near the research site. We will also have an arrangement with the Hospital de Trauma Senador Humberto Lucena for patient referral. Thus, this study will recruit patients with burn wounds residing in the State of Paraíba, who contacted researchers or were referred by the Burn Service of the Hospital de Trauma Senador Humberto Lucena, basic health units, or emergency care units of the city of João Pessoa (Paraíba, Brazil).

### Assignment of interventions: allocation

#### Sequence generation {16a}

The online software randomization.com will be used to allocate participants by blocks into three parallel groups (1:1:1) of 13 subjects: RLG, which will receive photobiomodulation using red LED; ILG, which will receive photobiomodulation using infrared LED; and SG, which will undergo a simulated application of LED therapy.

#### Concealment mechanism {16b}

The researcher responsible for randomization will forward sequentially numbered, opaque, and sealed envelopes to the therapist.

#### Implementation {16c}

Participants and assessors will be blind to allocation, which will be performed by an external researcher not directly involved with participants. The therapist will open the envelope containing the treatment for each participant before the session.

#### Assignment of interventions: blinding

##### Who will be blinded? {17a}

Participants, assessors, and data analysts will be blinded to treatment allocation. After assessments, assessors will fill in a questionnaire indicating which group the participant was allocated. Follow-up appointments will be scheduled to avoid overlap and communication among participants.

##### Procedure for unblinding if needed {17b}

Emergency unblinding will be allowed in case of withdrawal from the trial. Otherwise, to maintain the overall quality and legitimacy, the unblinding will occur exceptionally if the information is essential for further patient management.

### Data collection and management

#### Plans for assessment and collection of outcomes {18a}

Data will be collected using an evaluation form and the following tools: photographic and thermographic images, VAS, Itch Severity Scale, adheremeter, and Vancouver Scale. All standardized instruments used in the study procedure are described in the “Outcomes {12}” section.

#### Plans to promote participant retention and complete follow-up {18b}

Participants can leave the study at any time and without explanation. However, we will extensively inform them about the study setup and requirements during recruitment, highlighting the importance of a complete follow-up. During the study, participants will receive a phone call to remind and engage them before every consultation. In the follow-up period, researchers will verify responses and, if needed, contact participants to complete the follow-up (see the “Attrition and Adherence {11c}” section).

#### Data management {19}

Recruitment, randomization, and blind allocation will be conducted by a researcher who will not participate in other study stages. Experienced assessors will be responsible for recording data, while the project coordinator will be responsible for data management. Data will be collected in a printed form, exported to a digital spreadsheet (Excel®) with double typing and data validation, and stored in a file on the internet (cloud storage) by the project coordinator. Therapists will not have access to data.

#### Confidentiality {27}

All information obtained will be stored by the project coordinator at the Federal University of Paraíba. Individual trial identification numbers will be given to participants to ensure privacy regarding identity and data involved in the study. Researchers will also attest that the results obtained will be disclosed exclusively for academic purposes. Names of participants will only be recorded on the informed consent form, which will be kept untraceable in a locked cupboard with the project coordinator and separated from digital data. An anonymous database may be available in the future for collaborative research and prospective meta-analyses. Requests must be authorized by the principal investigator and human research ethics committees.

#### Plans for collection, laboratory evaluation, and storage of biological specimens for genetic or molecular analysis in this trial/future use {33}

Not applicable because no samples will be collected.

## Statistical methods

### Statistical methods for primary and secondary outcomes {20a}

Outcome variables will be re-epithelialization rate, presence and intensity of pain and pruritus, local skin temperature, and scar mobility and quality. Groups will be considered independent variables. Statistical analysis will be performed according to intention-to-treat principles. Descriptive analyses and histogram inspections will determine data normality. Between-group differences and 95% confidence intervals (95% CI) will be calculated using linear mixed models and group, time, and group-versus-time interaction terms. A first-order autoregressive covariance matrix will be considered. Group and time will be considered fixed factors, and participants the random factors. A Q-Q plot will be performed for all variables to consistently and similarly confirm the normality of residues between groups. The SPSS software version 20.0 (IBM SPSS Corp., Armonk, NY) will be used for data analysis.

### Interim analyses {21b}

An interim analysis will be performed on the primary endpoint after randomization and complete re-epithelization of 50% of participants. An independent and blinded statistician will perform the analysis and report to the independent data and safety monitoring committee (DSMC). The DSMC will have unblinded access to data and discuss the results with the steering committee. Then, the steering committee will decide on the trial continuation and report to the central ethics committee. The trial will end if any severe damage is identified in the experimental groups. The trial will not stop due to futility unless the DSMC advises otherwise during the course of safety monitoring. In this case, the DSMC will discuss the possible stop of the trial with the steering committee.

### Methods for additional analyses (e.g., subgroup analyses) {20b}

Not applicable. We do not plan to conduct subgroup or adjusted analyses.

### Methods in analysis to handle protocol non-adherence and any statistical methods to handle missing data {20c}

We propose an intention-to-treat analysis considering all participants, regardless of the randomized treatment. The intention-to-treat analysis will be applied using a mixed linear model, which treats missing data randomly.

### Plans to give access to the full protocol, participant-level data, and statistical code {31c}

The full protocol, pseudonymized dataset, and statistical codes will be available upon request after study publication.

### Oversight and monitoring

#### Composition of the coordinating center and trial steering committee {5d}

This single-centered trial will be supervised by the clinical research center and ethics committee of the Federal University of Paraíba. The principal investigator will coordinate the trial, and two researchers (one coordinator and one associate researcher) will ensure quality control according to the principle of good clinical practice. These two researchers will monitor the study progress, verify if the protocol is being followed, and submit the study status and review suggestions to the principal investigator, who will report to the ethics committee and organize discussions about the study protocol and data management. The principal investigator, the assessor, and the quality control coordinator will form the data management team, responsible for maintaining the database and confirming data accuracy, completeness, entry, and analysis.

#### Composition of the data monitoring committee, its role, and reporting structure {21a}

No data monitoring committee was appointed due to the low burden and minimal risks; therefore, researchers will be responsible for data monitoring. Periodically, the quality control team will randomly monitor the assessment and application of the intervention in all groups without prior notice.

#### Adverse event reporting and harms {22}

Aspects of the wound (e.g., color, temperature, and odor) and feelings of the participant regarding inconveniences during the session (e.g., pain) will be scored to control adverse events: no (1), mild (2), moderate (3), and strong (4). The number and management of adverse events and severity and duration of symptoms will also be recorded. The treatment will be discontinued if any damage or severe discomfort is identified.

#### Frequency and plans for auditing trial conduct {23}

Researchers will allow study-related monitoring, audits, and inspections by authorized organizations according to Good Clinical Practice guidelines. The following monitored aspects will be included: inclusion rate, informed consent progress, inclusion and exclusion criteria, trial master file, source data verification, safety reporting, trial procedures, and closing and reporting. Moreover, extensive auditing is not needed given the low risk of the intervention. Therefore, no audits are planned because the principal investigator will oversee all study activities.

Periodically, one researcher not involved in randomization, evaluation, and intervention will randomly select data from the evaluation form for auditing. This same researcher will also randomly monitor the assessment and application of the intervention in all groups without prior advice (see “Composition of the data monitoring committee, its role, and reporting structure {21a}” section).

#### Plans for communicating important protocol amendments to relevant parties (e.g., trial participants, ethical committees) {25}

Any protocol modification that may impact the study or benefit or endanger the participant will require a formal amendment to the protocol. These modifications include changes in study objectives, study design, population, sample size, study procedures, or significant administrative aspects. The research ethics committee will also be notified. This clinical trial protocol was prospectively registered in the Brazilian Clinical Trials Registry - ReBEC (RBR-8bfznx6). Changes in the protocol will also be registered in the ReBEC platform.

#### Dissemination plans {31a}

We will try to reduce to an absolute minimum the time between the end of data collection and release of study results in an appropriate journal; we expect a period between 4 and 5 months. Study results will be disseminated to the scientific community, physical therapists, participants, and the general medical community.

## Discussion

This study protocol is the first to compare the re-epithelialization rate of second-degree burns, pain, pruritus, skin temperature, quality of wound healing, and scar mobility among participants undergoing photobiomodulation using red and infrared LED. According to the randomization performed before recruitment, thirty-nine participants with burn wounds will be divided into three groups. The following variables will be assessed post-treatment and in the short term: re-epithelialization rate, skin temperature, pain intensity, pruritus intensity, and scar mobility and quality.

Changes in medication prescribed to participants will not be encouraged for ethical reasons, especially medication administered on demand to control symptoms. Both groups will be encouraged to perform standard care for their wounds.

Despite different therapeutic doses, a systematic review indicated that photobiomodulation improved burn healing in animals [23]. Furthermore, only one crossover clinical trial about the topic was published and presented methodological problems, such as the absence of blinding of therapist and participants [15, 16].

Regardless of wound type, the various parameters used in previous studies and clinical trials [24] hinder the recommendation of ideal doses and treatment procedures to heal burns. Because evidence is insufficient, the World Association for Photobiomodulation Therapy does not recommend treatment procedures and optimal doses.

Our study is a randomized controlled clinical trial with minimal risk of bias and intends to identify the type and doses of photobiomodulation that will improve the healing of burn wounds. Positive results will allow the recommendation and implementation of the technique in treatment protocols for these individuals.

## Trial status

This article is based on the study protocol version 1 of October 30, 2021. The study started on November 1, 2021. Participants are currently being recruited and enrolled; recruitment may continue until May 2024. Contact: Palloma Andrade, email: palloma@ccs.ufpb.br.

## Data Availability

The datasets used and analyzed during the study will be available upon request to the corresponding author after the publication of the results. Data regarding participant identification dispersed to the project team will be blinded to ensure confidentiality.

## Abbreviations

CI: Confidence Interval
ILG: Infrared light experimental group
IR: Infrared
LED: Light-emitting diode
ReBEC: Brazilian Clinical Trials Registry
RLG: Red light experimental group
SG: Sham group
SPIRIT: Standard Protocol Items: Recommendations for Interventional Trials
VAS: Visual analog scale
